# Reimagining statistical analysis for evidenced-based policy making: Early experiences using Stats Report

**DOI:** 10.7189/jogh.07.020306

**Published:** 2017-12

**Authors:** Emily Wilson, Lois A Park, Scott Zeger, Timothy Roberton

**Affiliations:** 1 Department of International Health, Johns Hopkins Bloomberg School of Public Health, Baltimore, Maryland, USA; 2 Department of Biostatistics, Johns Hopkins Bloomberg School of Public Health, Baltimore, Maryland, USA

Global health decision–makers need tools to more easily obtain, analyze, and use data. In the National Evaluation Platform (NEP), we worked with mid– and high–level government staff to build local capacity for data analysis. With these experiences came challenges in statistical capacity–building and collaboration that are likely to be similar in other contexts.

To address these challenges in the NEP, we used an online web application called Stats Report. Built on the R statistical package, Stats Report allows complex data analysis to be undertaken more easily and collaboratively. We used Stats Report in data analysis workshops with government staff in Malawi, Mali, Mozambique, and Tanzania. Statistical experts from our team prepared analysis code, and in–country workshop participants ran the analyses themselves, without having to adjust code, manipulate files, or download software. Using Stats Report, participants generated a variety of analytical outputs more quickly and more reliably than had been possible in previous workshops. We report the way in which participants used and responded to Stats Report.

Our early experiences suggest that Stats Report is easy to use, increases efficiency for data analysis, and enhances transparency and scientific replicability, which may be useful beyond the NEP. A unique strength is the ability to foster collaboration while encouraging users who first run existing analyses to later develop independent skills and the potential to teach others.

## BACKGROUND

Increasingly in global health, governments, donors, and the public are demanding that decisions be made in response to evidence [1]. For this to happen, decision–makers need skills and resources to obtain and interpret the results of scientific analyses. Yet many government policy–makers around the world, and even technical staff, have not been adequately trained in statistics or data science [2]. Often, analyses are outsourced to external experts who are not aware of the policy context, and not living where policies are implemented [3]. While external support can bridge capacity gaps, ultimately this further separates those who analyze from those who decide [4].

Through the National Evaluation Platform (NEP), the Institute for International Programs at Johns Hopkins University (IIP–JHU) has been supporting mid–level government staff in Malawi, Mali, Mozambique, and Tanzania to gather existing data, assess data quality, conduct statistical analyses, and report key messages to policy–makers [5]. The NEP emphasizes country–owned analyses; local institutions lead and conduct their own program and policy evaluations. A key part of this is building capacity in core statistical concepts so that national staff can analyze their data themselves. An independent evaluation of the NEP suggests increased commitment to high–quality data and scientific outputs among stakeholders [6]. However, in working with NEP members to do self–led data analysis, a variety of barriers came to the fore as we built statistical skills and computer competencies, critically thought about data, addressed software issues, and considered analytical documentation and replicability. Traditional education models for our capacity building purposes proved inadequate.

To help overcome these challenges in the NEP, we began using an online web application called Stats Report. Our initial experiences have been positive. Stats Report allows us to spend more time analyzing and interpreting data with workshop participants. Other policy–makers and scientific collaborators within government and non–governmental organizations may also benefit from using Stats Report. This paper describes our early use of Stats Report, and how it is enabling policy–makers to obtain analytical results more easily than before.

## STATS REPORT

Stats Report is an online web application, built on top of the R statistical package, for easy and quick data analysis. Users navigate to a website (http://statsreport.org) and select an analysis from a library of analyses, where an R script [7] and associated data are shared. Results are displayed and downloadable as graphs, tables, text, and maps. No software, other than the user’s web browser, needs to be installed on an individual’s computer in order to run analyses. The analyses that have been put into Stats Report, can be run online by anyone with access to the internet.

Users of Stats Report are largely either people who write R scripts and set up analyses to make their work accessible, or people who run analyses to obtain results and outputs (eg, statistical tests, tables, graphs, other visualizations). For the first group, Stats Report enables quick and wide dissemination and documentation of analyses, and a mechanism for easier collaboration with non–statistical colleagues. For the second group, Stats Report offers access to analyses that are statistically sound, which can be disseminated or revised. Stats Report facilitates learning and capacity building not only as workshop participants discuss results and decide what to do next with their outputs, but also by offering those who want to expand their analytical skills an opportunity to freely view, copy, edit, and learn from other users’ statistical scripts.

Stats Report currently hosts code which functions along the spectrum of data use, including code to: manage, analyze, visualize, share, teach, and collaborate. To manage data, users can merge, clean, and reformat data in a systematic way. To analyze data, users can perform summary or complex statistics, which may be customized through user–specified parameters. To visualize data, users can generate and observe outputs in Stats Report and download them. To collaborate, users can contribute to working versions of analyses, and run updates without worrying about versioning and IT issues. In all cases, users may use either pre–uploaded data to generate their outputs, or bring their own new data to existing analyses.

Photo: Workshop on Stats Report from Mozambique. From the collection of Talata Sawadogo-Lewis (used with permission).

## COUNTRY–LEVEL USE OF STATS REPORT

As part of the NEP, we conducted multiple data analysis workshops with government staff in Malawi, Mali, Mozambique, and Tanzania. The purpose of these workshops was to analyze local data in response to NEP questions and, at the same time, to increase capacity of the NEP members in attendance. NEP teams within the four countries varied in size (Malawi: 40, Mali: 11, Mozambique: 36, Tanzania: 27), as well as sex ratio, age, and technical ability. These people have backgrounds as health workers, managers, statisticians, economists, IT personnel, researchers, lecturers, and professors. Our goal was to train these colleagues to asses and utilize data for maternal and child health and nutrition program evaluation. In collaboration, we assessed data quality, calculated coverage of interventions, analyzed lives saved due to health interventions, demonstrated statistical concepts such as uncertainty and regression using country data, and applied advanced statistical concepts to questions generated by in–country colleagues.

Initially we tried teaching workshops using Stata, and sharing code and data on secure cloud storage. While this met some objectives, we found that file naming conventions, and folder permissions did not foster institutional knowledge transfer, and some colleagues found accessing and updating analyses challenging. It was difficult for colleagues to rerun an analysis, if a software version or operating system on their computer differed from the one that originally generated the analysis. Additionally, we found that formatting and defaults associated with French and Portuguese accented labelling, for example, were not easy to standardize or maintain on individuals’ computers. As the NEP incorporated more statistical work, generating results became time–consuming.

In mid–2016, we began using Stats Report, which has addressed many of these challenges. Stats Report has allowed us to teach workshops with fewer time–taking IT tasks, which can interrupt workshop learning. Outputs appear reliably on all participants’ computers, the same way that the analyst prepared them. We can disseminate workshop material more efficiently. If someone encounters a bug in an analysis, we can fix it one time, in real–time, and have participants reload statsreport.org and proceed, rather than waiting until a new version of the code has been installed on everyone’s computer. If participants request changes to an analysis, we can implement changes and share updates on Stats Report. Furthermore, R is free, so software licensing is not a concern.

In Mozambique, country stakeholders participated in a workshop to analyze routine data. Participants from the Ministry of Health came to the workshop with data extracted from the national health management information system (HMIS). A statistician prepared simple scripts that graphed variables by month or year, with a linear or polynomial fitted curve overlaid to show the trend over time. Participants uploaded data, selected the analysis, and chose the columns within their data that corresponded to the variables needed for the underlying code to produce trends. Participants generated complex graphs and discussed, downloaded, shared, and copied statistical results into written policy briefs and technical reports. Workshop participants appreciated the ability to generate customized results without needing to adjust code themselves. “The main [advantage] for me is the fact that we don't need to be expert in R language to use Stats Report, the program allows us to do complex analyses in a much easier way” [8].

Members of the Mali task team participated in a workshop on regression. Stats Report was used to illustrate basic principles of linear regression, as well as to prepare complex data sets and results. Workshop participants first used the simple examples to learn about regression, and later furthered their country–led analysis by obtaining reproducible outputs from more advanced statistical scripts. These participants then spent time meaningfully interpreting their results. Capacity building workshops in the NEP prior to Stats Report had necessarily focused more time during workshops on simply obtaining outputs. As one participant said, “[the advantage of Stats Report is it’s] easy to use, and orientation to different results is fast. One can obtain estimates, confidence intervals, *P*–values, odds ratios, tables and graphs” [8].

Outside of workshops, Stats Report has fostered collaboration and built capacity by providing a way to make results available to countries continuously. Stakeholders in NEP countries request analyses that are written by an IIP statistician. In–country technical teams run the analyses independently, download the generated outputs, and use these results in reports, and as figures in presentations to high–level government stakeholders.

While Stats Report has undoubtedly helped us in our capacity building workshops, there are ways to improve it. Workshop participants have mentioned the need for trainings or guidance, and we recognize that we have had more success introducing Stats Report where new users are given repeated opportunities to interact with it, before incorporating it into their work.

## A WAY FORWARD FOR COLLABORATIVE DATA ANALYSIS

Our experiences suggest that Stats Report is able to aid global health practitioners by allowing them to run their analyses without knowing a statistical programming language, the same way one can drive a car without knowing how the engine works.

**Figure 1:**
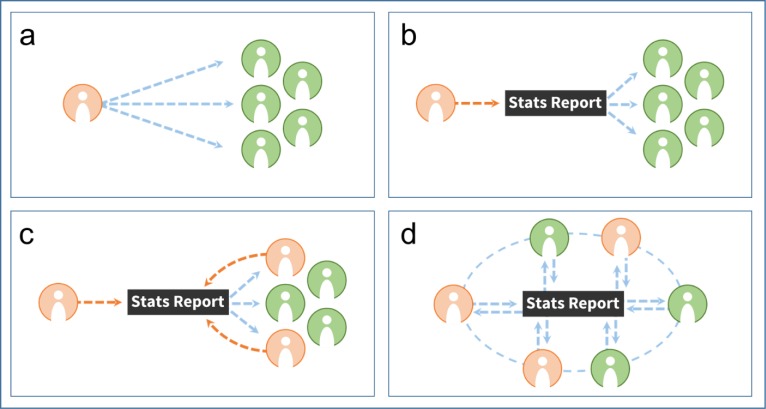
a) traditional model of capacity building, b) capacity building using Stats Report, c) code users becoming code contributors, and d) data analysis in a network of users.

We started the NEP with a capacity building strategy in which an external facilitator teaches concepts and methods to a group of people (panel a in **Figure 1**). With the advent of Stats Report, a statistician now puts results into Stats Report and workshop participants use Stats Report to generate results for interpretation and discussion (panel b in **Figure 1**). As individuals further develop skills and analyses, they can contribute code for others to use (panel c in **Figure 1**). We hope that Stats Report eventually develops into a community of people who both use and contribute code (panel d in **Figure 1**).

In our experience, the situation in panel a **Figure 1** is often a one–time event, with poor documentation and little hope for sustainability. Panel b in **Figure 1** has facilitated better short–term results than traditional capacity building, and fosters collaboration between multi–disciplinary teams between workshops, which is conducive to longer–term capacity building. The capacity jump from the situation in panel b to that in panel c in **Figure 1** is sizable, however, Stats Report involves the documentation of code, and making results reproducible and accessible such that panel c in **Figure 1** represents what we see starting to happen, when individuals learn to edit existing R code to produce different or additional outputs. Panel d in **Figure 1** shows how we think workshop participants could collaborate beyond the workshop, in the future. A network of advanced users who openly share analytical solutions could find faster and better answers to maternal and child health and nutrition program questions.

## CONCLUSION

To further promote evidenced–based decision–making in global health, we need more accessible and intuitive ways to analyze and use data. We need tools that require minimal training, so people with limited skills can quickly generate valid results, and so institutional capacity is not lost when trained staff leave. Meanwhile, we need people with advanced skills to generate and share results that can be tailored so others can also benefit. When external statistical experts are employed, we need more effective collaboration that builds long–term capacity without creating dependence.

Within the NEP, Stats Report has addressed these needs by providing a user–friendly means for our US–based staff and in–country partners to collaborate. We believe others would similarly benefit from using Stats Report. It helps people analyze data more easily, which is imperative for evidenced–based policy making. It helps people to both run an analysis immediately, and learn from it later. In our experience, Stats Report has been an invaluable way for policy–makers, government staff, and collaborators to better analyze data, and to produce greater evidence.

## References

[1] Victora C, Requejo J, Boerma T, Amouzou A, Bhutta ZA, Black RE (2016). Countdown to 2030 for reproductive, maternal, newborn, child, and adolescent health and nutrition.. Lancet Glob Health.

[2] Adam T, Ahmad S, Bigdeli M, Ghaffar A, Rottingen JA (2011). Trends in health policy and systems research over the past decade: still too little capacity in low-income countries.. PLoS One.

[3] Deloitte US Strategy and Operations. Avoiding common outsourcing pitfalls. Available: https://www2.deloitte.com/us/en/pages/operations/articles/avoiding-common-outsourcing-pitfalls.html. Accessed: 5 April 2017.

[4] Boerma T, Mathers CD (2015). The World Health Organization and global health estimates: improving collaboration and capacity.. BMC Med.

[5] Victora CG, Black RE, Boerma JT, Bryce J, Bryce J, Victora C (2011). Measuring impact in the Millennium Development Goal era and beyond: a new approach to large-scale effectiveness evaluations.. Lancet.

[6] FSG. National Evaluation Platform: Findings from the Midpoint Evaluation. 2015.

[7] R Core Team. R: A language and environment for statistical computing. 2014. Available: http://www.r-project.org. Accessed: 16 March 2016.

[8] Sawadogo-Lewis TJ. Stats Report User Experience [Google form]. Available: https://docs.google.com/forms/d/16RGAWPIOQ93XEAO3gtIozLnabJWCDrrDOLDjBnlv6F0/edit?ts=599436c9#responses. Accessed: 16 March 2016.

